# The Link Between Dietary Timing and Exercise Performance Through Adipocyte AMPKα2 Signaling

**DOI:** 10.3390/ijms26136061

**Published:** 2025-06-24

**Authors:** Sohyun Kim, Jihyun Baek, Man S. Kim

**Affiliations:** 1 Translational-Transdisciplinary Research Center, Clinical Research Institute, Kyung Hee University Hospital at Gangdong, College of Medicine, Kyung Hee University, Seoul 05278, Republic of Korea; ppeach3@khu.ac.kr; 2 Department of Medicine, College of Medicine, Kyung Hee University, Seoul 02453, Republic of Korea; 3 Division of Nephrology, Department of Internal Medicine, CHA Bundang Medical Center, CHA University, Seongnam 13496, Republic of Korea; spreesh7@chamc.co.kr

**Keywords:** adipocyte AMPKα2 signaling, circadian rhythms, day-restricted feeding, fat–muscle crosstalk, exercise timing, metabolic health, multi-omics, chronopharmacology, time-restricted feeding, mesenchymal stem cells

## Abstract

Emerging evidence suggests that the timing of eating and exercise over the course of the day is paramount to metabolism and physical function. This review highlights seminal studies showing that adipocyte AMPKα2 signaling controls circadian adipose tissue–skeletal muscle communication. Day-restricted feeding has been shown to improve exercise performance via adipocyte-specific activation of AMPKα2, which controls fat–muscle crosstalk in a time-of-day dependent manner. This review also discusses corroborating experimental studies designating mesenchymal stem cells as key cellular mediators, showing that exercise in the afternoon leads to better metabolic effects in humans, and illustrating how incorrect timing of food intake leads to leptin resistance and metabolic dysregulation. Multi-omics strategies have shed light on the molecular mechanisms underlying such effects of time, showing the circadian control of metabolic processes across tissues. These results advance our knowledge of chronometabolism and offer exciting temporal intervention treatments for metabolic diseases, such as time-restricted feeding, timed exercise, and chronopharmacological targeting of AMPK. Fat–muscle crosstalk, physical performance, and metabolic health outcomes can possibly be optimized by synchronizing dietary and exercise timing with endogenous circadian rhythms.

## 1. Introduction

The global obesity and metabolic disease epidemic represents one of the greatest public health challenges of our time. The World Health Organization reports that over 1.9 billion adults globally are overweight, with obesity affecting more than 650 million of them [1]. While traditional caloric restriction and exercise interventions are mainstay therapies, evidence is building that the timing component of these interventions—more specifically ‘when’ we eat and exercise—can be as critical as ‘what’ and ‘how much’ [2].

Circadian rhythms govern nearly all physiological processes over a 24 h cycle, and their disruption is causally linked to an increased risk of insulin resistance, obesity, and metabolic syndrome [3]. This temporal structuring of metabolism is not simply a result of behavioral patterns but is a manifestation of basic molecular processes that coordinate energy homeostasis between tissues. Despite this knowledge, the vast majority of research and clinical practice today overlook the temporal dimension of metabolic interventions, which is a critical gap in our strategy for managing metabolic diseases.

Classically, exercise physiology research has adopted a muscle-centric paradigm, and the adipose tissue has been viewed as a relatively passive energy storage organ. However, emerging evidence indicates that adipose tissue is a highly dynamic endocrine organ that potentially plays a role in coordinating intertissue communication and exercise performance through circadian-related processes [4]. This paradigm has been compelled by recent technological advances, particularly multi-omics approaches, which have revealed complex molecular networks of tissue–tissue communication.

Previous reviews in the field have tended to cover either circadian metabolism or exercise–adipose tissue interactions independently. However, the convergence of these two fields—temporal regulation and adipose muscle communication—remains largely unexplored. Most existing reviews do not include a detailed discussion of how timing interventions actually control the molecular mechanisms of fat–muscle communication, particularly through new pathways such as adipocyte AMPKα2 signaling.

Novel seminal research by Chen et al. (2025) revealed adipocyte AMPKα2 signaling as a master regulator of time-dependent fat–muscle crosstalk, demonstrating that dietary timing can enhance exercise performance through this novel pathway [5]. This is a quintessential example of how temporal interventions can leverage endogenous circadian mechanisms to enhance metabolic health, a paradigm shift from conventional approaches, whereby timing is secondary to intervention content.

The purpose of this review is to synthesize the current understanding of how adipocyte AMPKα2 signaling orchestrates the timing of exercise and diet execution. We aimed to present a detailed exploration of the molecular foundations of this interaction based on cutting-edge multi-omics studies and to translate the findings into clinically relevant temporal intervention strategies. In particular, we will (1) review the circadian regulation of adipose tissue metabolism and its role in inter-tissue communication; (2) delineate the tissue-specific actions of AMPKα2, including its newly revealed role in adipocytes; (3) untangle the molecular mechanisms of fat–muscle communication and how timing affects this communication; (4) integrate understanding on the interaction between dietary timing and exercise at both the mechanistic and clinical levels; (5) underscore how multi-omics approaches have revolutionized understanding of these temporal metabolic networks; and (6) translate these insights into immediate clinical practice and future research directions.

By integrating the overlap between circadian biology, exercise physiology, and clinical practice, this review aimed to establish a basis for the development of evidence-based temporal intervention strategies that leverage the therapeutic potential of circadian rhythms to enhance metabolic health and physical performance. The integration of timing in metabolic medicine represents not a gradual advance but rather a fundamental reconsideration of the prevention and treatment of metabolic diseases.

## 2. Summary of Key Studies on Temporal Aspects of Fat–Muscle Crosstalk

Several recent studies have highlighted the importance of temporal factors in metabolism. Yang et al. (2022) used a single-cell analysis to investigate the effects of obesity and exercise on adipose muscle tissues by targeting mesenchymal stem cells and circadian rhythm-related pathways [6]. Their investigation revealed that stromal cells, such as mesenchymal stem cells, undergo extreme circadian gene alterations in response to obesity and exercise, outlining the cellular basis of fat–muscle circadian crosstalk. Specifically, extracellular matrix remodeling and circadian rhythm emerged as the most prominent pathways regulated by exercise and a high-fat diet in mesenchymal stem cells across multiple tissues [6].

Savikj et al. (2022) investigated the influence of exercise timing on multitissue metabolome and skeletal muscle proteome profiles in patients with type 2 diabetes, demonstrating that afternoon exercise significantly enhanced muscle lipid and mitochondrial content compared to morning exercise [7]. This finding has been supported by additional studies examining exercise timing effects. Mancilla et al. (2021) demonstrated that afternoon exercise training resulted in superior metabolic benefits compared to morning training in metabolically compromised subjects, including improved peripheral insulin sensitivity and exercise performance [8]. Similarly, Iwayama et al. (2021) found that diurnal variations in muscle and liver glycogen differ significantly depending on exercise timing [9].

Recent investigations have also explored the molecular mechanisms underlying exercise timing effects. Ezagouri et al. (2019) revealed that both mice and humans exhibit daytime variance in exercise capacity, which depends on circadian clock proteins PER1/2 and involves time-dependent activation of ZMP, an endogenous AMPK activator [10]. This temporal regulation of exercise performance appears to be mediated through specific metabolic pathways that are differentially activated based on the time of day [10].

Oishi and Hashimoto (2018) revealed that time-restricted feeding, when implemented during the resting phase, leads to leptin resistance, thereby promoting obesity and metabolic disorders [11]. This study established that an acute misalignment of feeding time with the circadian cycle results in profound metabolic dysregulation. Basse et al. (2018) showed that skeletal muscle insulin sensitivity is marked by circadian rhythmicity, irrespective of exercise training status [12], indicating that the circadian regulation of muscle metabolism is mediated via mechanisms distinct from those caused by exercise adaptation.

Additional research has focused on the cellular mechanisms of temporal metabolic regulation. Noshiro et al. (2020) demonstrated that DEC1 regulates the rhythmic expression of PPARγ target genes involved in lipid metabolism in white adipose tissue, showing that circadian transcriptional control extends to specific metabolic pathways [13]. Furthermore, Ma et al. (2024) revealed that disruption of the BMAL1/REV-ERBα circadian rhythmic loop is associated with excessive fat expenditure in heart failure, highlighting the clinical significance of maintaining circadian timing in metabolic health [14]. These representative studies are summarized in Table 1, highlighting key findings from both animal and human models on how temporal factors influence fat–muscle communication.

## 3. Circadian Rhythms and Adipose Tissue Metabolism

Circadian rhythms, virtually 24 h cycles that govern physiology, play a critical role in the metabolism of adipose tissue [15]. These biological rhythms are governed by a complex network of molecular clocks. The suprachiasmatic nucleus (SCN), located in the hypothalamus, acts as the master clock, coordinating the peripheral clocks found in various tissues, including adipose tissue [16]. The underlying mechanism of this molecular clock is a feedback loop involving core clock genes like Clock, Bmal1, Per, and Cry, which in turn regulate the expression of hundreds of downstream genes critical for metabolic processes [17].

The adipose tissue has also been found to be an extremely active organ in circadian regulation. This tissue shows robust diurnal rhythms of gene expression, with a large number of genes exhibiting circadian expression patterns [15]. These genes are involved in other metabolic processes, including lipogenesis, lipolysis, adipogenesis, and thermogenesis. The circadian regulation of adipose tissue extends beyond gene expression to protein abundance, enzyme activity, and metabolite levels.

Chen et al. (2025) outlined a broad picture of how day-restricted feeding influences the diel rhythms of most molecular components of adipose tissue [5]. By utilizing cutting-edge multi-omics profiling, these researchers demonstrated that daily restricted feeding (DRF) regulates the diel patterns of the mitochondrial proteome, neutral lipidome, and nutrient-sensing networks within mouse gonadal white adipose tissue (GWAT) [5]. Their research revealed that DRF significantly enhanced the amplitude of circadian rhythms in metabolic proteins and genes, suggesting that feeding time can render the molecular clock more robust in the adipose tissue.

The molecular mechanisms underlying circadian control of adipose tissue metabolism involve multiple transcriptional regulators. Noshiro et al. (2020) demonstrated that DEC1, a basic helix-loop-helix transcriptional repressor, interacts with the PPARγ:RXRα heterodimer to suppress transcription from PPARγ target genes in white adipose tissue [13]. They found that PPARγ target genes responsible for lipid metabolism, including triacylglycerol synthesis and lipolysis, oscillate in a circadian manner, and DEC1 deficiency disrupts these rhythms [13]. Similarly, Kadiri et al. (2015) showed that the nuclear retinoid-related orphan receptor-α (RORα) regulates adipose tissue glyceroneogenesis in addition to hepatic gluconeogenesis, demonstrating another layer of circadian metabolic control [18].

The time course of the expression of metabolic genes in adipose tissue has physiological consequences on the overall body metabolism. For instance, genes involved in fatty acid synthesis are upregulated when an animal is fed, whereas genes involved in fatty acid oxidation and lipolysis are upregulated when the animal is fasted [19]. The time course of this regulation enables the adipose tissue to store energy in times of abundance and release energy in times of stress.

The metabolic actions of circadian disruption in adipose tissue have been demonstrated in various experimental models. Recent research has shown that circadian disruption affects different types of adipose tissue distinctly. Dhamrait et al. (2020) found that exposure to low-dose ultraviolet radiation suppresses diet-induced obesity through circadian-dependent mechanisms involving brown adipose tissue function [20]. Their study revealed that circadian modulation affects UCP-1 expression and prevents the “whitening” of brown adipose tissue induced by high-fat diet consumption [20].

Recent studies have detailed the mechanistic relationships between circadian disruption and dysregulation of adipose tissue. Paschos et al. (2012) demonstrated that adipocyte-specific Arntl (Bmal1) knockout resulted in obesity and metabolic disease [21]. This suggests that peripheral clocks in adipose tissue are not merely passive recipients of signals from the central clock, but rather play an active role in metabolic regulation. Loss of the clock in adipocytes results in the dysregulation of lipid metabolism, increased adiposity, and impaired glucose tolerance.

The adipose tissue metabolic timing at feeding time is mediated, at least in part, by adipocyte AMPKα2 signaling [5]. Chen et al. (2025) showed that specific adipocyte knockdown of Prkaa2 resulted in arrhythmicity of genes involved in acyl-CoA metabolism and loss of rhythmicity in the GWAT lipidome [5]. This suggests that AMPKα2 is a critical molecular link between nutrient status and the circadian clock of the adipose tissue. Protein kinases appear to integrate information regarding energy availability with temporal information to control metabolic processes in the adipose tissue.

Notably, circadian regulation of adipose tissue metabolism has been observed to extend to different adipose depots. White, brown, and beige adipose tissues exhibit diverse circadian rhythms in gene expression and metabolic activity [22]. For example, brown adipose tissue is augmented in thermogenic gene expression and metabolic activity during the active portion of the day, whereas white adipose tissue demonstrates increased lipolysis during fasting.

The clinical relevance of circadian disruption in adipose tissue metabolism has been demonstrated in disease models. Ma et al. (2024) showed that in heart failure rats, excessive fat expenditure is associated with disruption of the BMAL1/REV-ERBα circadian rhythmic loop [14]. Their study revealed that disrupted circadian circuits in adipose tissue led to increased lipolysis, enhanced brown adipose tissue thermogenesis, and elevated plasma inflammatory markers [14]. This work highlights how pathological conditions can disrupt adipose tissue circadian function with systemic metabolic consequences.

The two-way interplay between circadian rhythms and adipose tissue metabolism extends beyond the local level and regulates whole-body energy homeostasis [21]. Adipose tissue interacts with other organs, including the liver, muscles, and brain, by releasing a group of signaling molecules whose synthesis and release are controlled by circadian rhythms [22]. Adipokines such as adiponectin, leptin, and resistin have diurnal rhythms in their release and are crucial for coordinating the metabolism among tissues.

Dissociation of this timing coordination can lead to metabolic dysregulation in many tissues and cause systemic metabolic diseases such as obesity and type 2 diabetes [3]. For instance, shift work that interferes with normal feeding times and sleep–wake cycles is associated with the risk of metabolic syndrome, obesity, and type 2 diabetes. These relationships emphasize the importance of being in the proper circadian alignment in metabolic health. These findings collectively underscore the need for temporal coordination between fat and the circadian clock to maintain metabolic health.

## 4. Tissue-Specific Roles of AMPKα2 Signaling

AMPK, a highly conserved serine/threonine kinase, functions as a cellular energy sensor, maintaining energy homeostasis by responding to fluctuations in the AMP/ATP ratio [23]. This enzyme is a heterotrimeric protein comprising a catalytic α subunit and regulatory β and γ subunits [24]. The structural organization of this complex is critical for its function, with each subunit contributing distinct properties to the overall enzyme activity. In mammals, the catalytic α subunit exists as two isoforms, AMPKα1 and AMPKα2, which are encoded by the Prkaa1 and Prkaa2 genes, respectively [25]. The β subunit has two isoforms (β1 and β2), and the γ subunit has three isoforms (γ1, γ2, and γ3), allowing for the formation of at least 12 different AMPK complexes with distinct biochemical fingerprints and tissue expression patterns [26].

The β subunit contains several critical functional domains, including an N-terminal myristoylation site that facilitates membrane association, a conserved carbohydrate-binding module (CBM) that enables interaction with glycogen, and a C-terminal domain that mediates association with the α and γ subunits [27]. The γ subunit provides the energy-sensing functionality of AMPK through its four cystathionine-β-synthase (CBS) domains that create two Bateman domains capable of binding adenine nucleotides [28]. The CBS1 and CBS3 domains appear to be the primary sites responsible for energetic sensing, with CBS3 serving as the major allosteric regulatory site.

Although the α1 and α2 isoforms are functionally redundant and highly homologous, recent evidence suggests distinct tissue-specific functions and regulatory mechanisms for each isoform [23]. AMPKα1 is ubiquitously expressed across most tissues, while AMPKα2 shows a more restricted expression pattern, being expressed at high levels in skeletal and cardiac muscle, where it is the dominant α-subunit, and in the liver, while being found at lower levels in other tissues including adipose tissue [29]. The biochemical properties of α1- and α2-containing complexes also differ: baseline activity of α1-AMPK is generally higher than its α2 counterpart, but α2 complexes demonstrate a greater response to energy stress and appear to be better substrates for upstream kinases such as LKB1 [30]. Additionally, α2-containing complexes exhibit preferential nuclear localization compared to α1 complexes, and the two isoforms show slightly different substrate preferences [31].

The discovery of tissue-specific functions of AMPKα2 has revolutionized our knowledge of AMPK signaling. AMPKα2 is the predominant isoform in skeletal muscle and is responsible for crucial functions including contraction-stimulated glucose uptake, fatty acid oxidation, and mitochondrial biogenesis [32]. Activation of AMPK in muscles during exercise causes the phosphorylation and inhibition of acetyl-CoA carboxylase (ACC), inhibiting the synthesis of fatty acids and promoting fatty acid oxidation. AMPK also phosphorylates and activates PGC-1α, a transcriptional coactivator that plays a pivotal role in regulating mitochondrial biogenesis, thereby enhancing muscle oxidative capacity [33]. The role of AMPKα2 in muscle glucose uptake involves phosphorylation of TBC1D1 and TXNIP, which control the translocation and cell-surface levels of glucose transporters GLUT4 and GLUT1, respectively [34].

In liver, AMPKα2 regulates gluconeogenesis and lipogenesis according to nutritional status [35]. During fasting, hepatic AMPK activation represses ACC and SREBP-1c expression, thereby inhibiting fatty acid synthesis and promoting fatty acid oxidation. AMPK also inhibits and phosphorylates HMG-CoA reductase, which is a crucial regulatory enzyme in cholesterol biosynthesis, thus connecting it to the maintenance of lipid homeostasis [35].

However, the role of AMPKα2 in adipose tissue has been less clearly established until recently. The seminal article by Chen et al. [5] presented strong evidence for a specific function of adipocyte AMPKα2 in modulating physical endurance and fat–muscle crosstalk [5]. Using an adipocyte-specific Prkaa2 knockdown model, they showed that the decreased expression of AMPKα2 specifically in adipocytes compromised physical endurance in mice. This deficiency has been associated with disrupted rhythmicity in acyl-coenzyme A (CoA) metabolism genes, a disruption of the GWAT lipidome’s inherent rhythmicity, and remodeling of serum metabolites along a circadian axis.

The most striking finding was that adipocyte Prkaa2 depletion affected clock gene expression in muscle under day-restricted feeding (DRF) conditions, which reveals a novel pathway of inter-organ communication via adipocyte AMPKα2. These results challenge the general view of AMPK as a cell-autonomous energy sensor and reveal its role in coordinating metabolism across tissues. In order to determine the mechanisms by which adipocyte AMPKα2 influences muscle clock genes, Chen et al. (2025) undertook thorough molecular profiling [5]. They found that the adipocyte-specific Prkaa2 knockout caused extensive changes in the GWAT lipidome, significantly influencing the rhythmic patterns of acyl-CoA species. These lipid metabolites have the potential to function as signaling molecules or metabolic substrates that influence gene expression in distant organs.

The tissue-specific functions of AMPKα2 in adipose tissue extend beyond sensing energy to include regulation of adipogenesis, lipolysis, and secretion of adipokines [36]. In adipocytes, AMPK activation inhibits lipogenesis by phosphorylating ACC, and induces lipolysis by stimulating hormone-sensitive lipases [37]. AMPK also regulates the expression and secretion of adiponectin, a key adipokine that enhances insulin sensitivity in the muscles and liver [38]. Current studies have also revealed that AMPKα2 in the adipose tissue plays a role in the browning of white adipose tissue [39]. Exercise-induced activation of AMPK in adipocytes induces browning gene expression, which subsequently leads to improved thermogenic capability of white adipose tissue.

Basse et al. (2018) examined the circadian rhythmicity of insulin sensitivity in skeletal muscles and reported that basal and insulin-stimulated glucose uptake, along with insulin-stimulated pathways of glucose uptake such as AMPK phosphorylation, exhibit a diurnal rhythm [12]. Interestingly, exercise training increases skeletal muscle glucose uptake but does not influence the circadian rhythmicity of insulin sensitivity, indicating that the circadian regulation of muscle metabolism is independent of acute exercise effects [12]. This finding suggests that the circadian control of AMPK signaling in muscles occurs through mechanisms distinct from exercise-stimulated AMPK.

**Figure 1:**
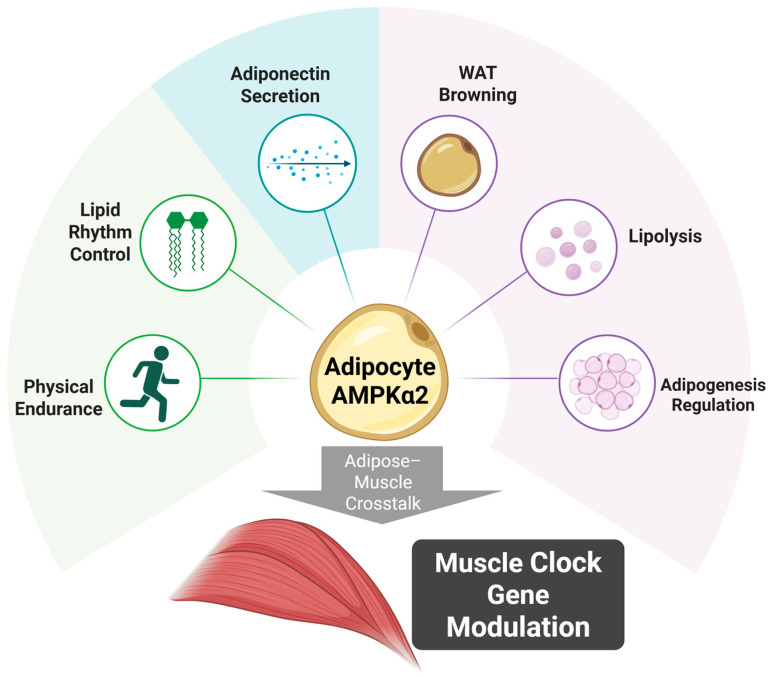
Role of adipocyte AMPKα2 in muscle clock gene modulation.Adipocyte AMPKα2 serves as a central regulator of adipose tissue metabolism and inter-organ communication. It modulates lipid rhythmicity, enhances physical endurance, and promotes adiponectin secretion, white adipose tissue (WAT) browning, lipolysis, and adipogenesis regulation. Through these mechanisms, adipocyte AMPKα2 exerts downstream effects on skeletal muscle, contributing to the modulation of muscle clock gene expression and maintaining circadian alignment across tissues. This model illustrates how AMPKα2 activity in adipocytes integrates nutrient status and energy demand to coordinate systemic metabolic timing.

The divergent activities of AMPKα2 in different tissues are a reflection of each tissue’s own metabolic demands and regulatory mechanisms [35]. For example, skeletal muscle requires instantaneous activation of AMPK to deal with transient deficiencies in energy that occur during exercise, whereas adipose tissue uses AMPK for more complex activities, such as long-term metabolic control, circadian regulation coordination, and inter-tissue communication [40]. The multiplicity of AMPK activity in different tissues provides the metabolic complexity necessary for coordination between organs. This concept is illustrated in Figure 1, which summarizes the multiple metabolic outputs of adipocyte AMPKα2 and its downstream impact on muscle clock gene regulation.

## 5. Molecular Mechanisms of Fat–Muscle Crosstalk

Bidirectional communication between the skeletal muscle and adipose tissue, or fat–muscle crosstalk, is essential for maintaining metabolic homeostasis [39]. Complex interactions involve the exchange of countless signaling molecules, including adipokines, myokines, metabolites, and extracellular vesicles, which orchestrate energy metabolism, insulin sensitivity, and physical performance [41]. Novel insights into the molecular mechanisms of this crosstalk have emerged from recent experimental studies, highlighting adipocyte AMPKα2 signaling as the central regulator.

Chen et al. (2025) made a significant contribution to our understanding of fat–muscle crosstalk by demonstrating that adipocyte AMPKα2 regulates muscle clock genes under day-restricted feeding (DRF) [5]. This study identified a novel pathway whereby adipose tissue signals influence gene expression in skeletal muscles in a circadian manner [5]. Specifically, they found that adipocyte-specific Prkaa2 knockdown led to alterations in clock gene expression in skeletal muscle, showing that adipocyte AMPKα2 signaling is crucial for proper muscle clock functioning under DRF [5].

### 5.1. AMPKα2-Mediated Regulation of Core Clock Genes

The mechanistic basis of adipocyte AMPKα2’s influence on muscle clock genes involves direct and indirect regulation of core circadian transcriptional machinery. AMPK has been established as a critical regulator of circadian clock proteins through post-translational modifications that affect their stability and function [42]. The core mammalian circadian clock is based on a transcriptional–translational feedback loop where the transcription factors CLOCK and BMAL1 form a heterodimeric complex that activates the expression of their own repressors, the period (PER1, PER2, PER3) and cryptochrome (CRY1, CRY2) proteins [43].

AMPK directly phosphorylates CRY1 and CRY2 at specific serine residues, targeting them for ubiquitination and proteasomal degradation [44]. This phosphorylation occurs at evolutionarily conserved sites: serine 71 and serine 280 in CRY1, which are critical for CRY1 stability. When AMPK activity is enhanced, as occurs during energy stress or nutrient deprivation, increased CRY1 phosphorylation leads to enhanced degradation of this transcriptional repressor. The destabilization of CRY1 relieves the inhibition of CLOCK/BMAL1 complexes, allowing for increased transcriptional activity of clock-controlled genes.

The regulation extends to PER proteins through an indirect mechanism. AMPK phosphorylates casein kinase 1ε (CK1ε), increasing its kinase activity [45]. Active CK1ε then phosphorylates PER proteins, promoting their degradation and further reducing the repressive capacity of the PER/CRY complex. This dual targeting of both CRY and PER proteins by AMPK-dependent phosphorylation provides a robust mechanism for energy-dependent regulation of circadian gene expression.

BMAL1, the positive transcriptional regulator, is also subject to AMPK-mediated regulation, though through more complex mechanisms involving NAD+-dependent pathways [46]. AMPK-induced NAD+ elevation enhances SIRT1 activity, which modulates chromatin states at clock gene loci. This creates a metabolic–circadian coupling where cellular energy status directly influences the amplitude and phase of circadian oscillations.

### 5.2. Adipocyte-to-Muscle Signaling Through Metabolic Intermediates

**Figure 2:**
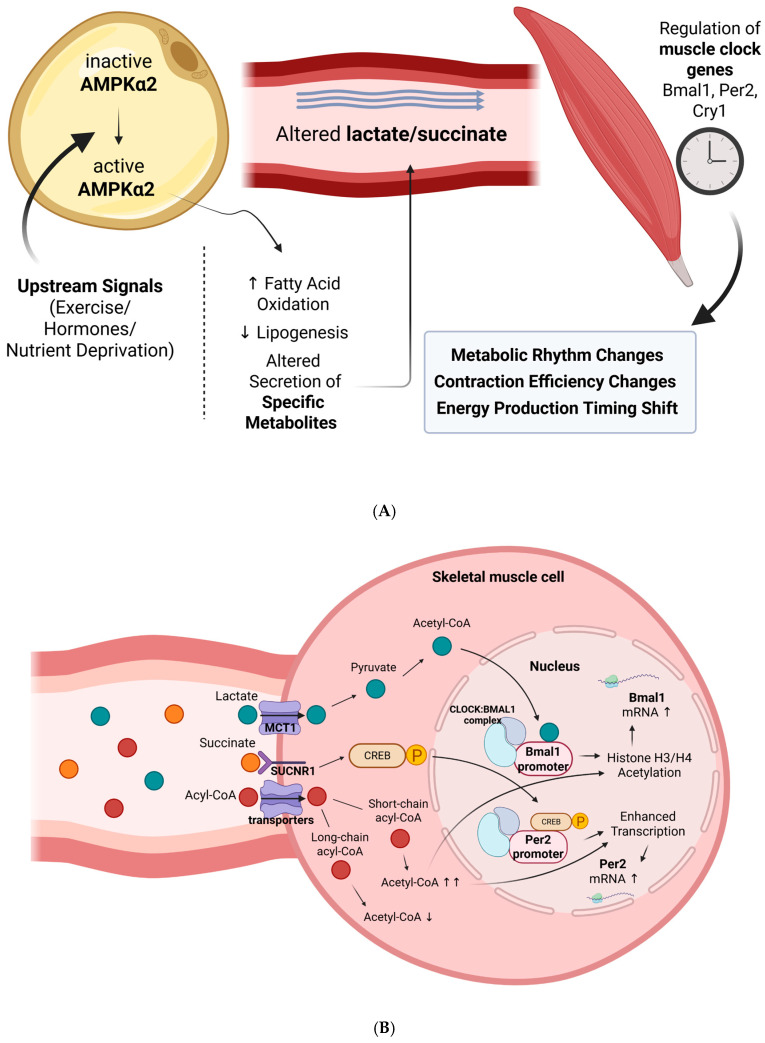
(**A**). Systemic regulation of muscle clock genes by adipocyte AMPKα2 signaling. Activation of adipocyte AMPKα2 by upstream signals (e.g., exercise, hormones, nutrient deprivation) enhances fatty acid oxidation and suppresses lipogenesis, leading to altered secretion of metabolites such as lactate and succinate. These circulating metabolites act on skeletal muscle, modulating clock gene expression (e.g., Bmal1, Per2, Cry1) and shifting metabolic rhythms, contraction efficiency, and energy production timing. This pathway illustrates the role of adipocyte-derived time-sensitive metabolic signals in synchronizing muscle circadian functions, particularly under day-restricted feeding conditions. (**B**). Intracellular mechanisms linking circulating metabolites to clock gene activation in skeletal muscle. Schematic illustration shows how day-restricted feeding (DRF) activates adipocyte AMPKα2, leading to the secretion of metabolic intermediates that regulate muscle clock genes. Cytoplasm: Circulating lactate enters muscle cells via MCT1 transporter and is converted to acetyl-CoA. Succinate binds to SUCNR1 receptor, activating a signaling cascade that phosphorylates CREB. Acyl-CoA species are transported into cells via multiple transporters, altering cellular acetyl-CoA availability. Nucleus: At the Bmal1 promoter, CLOCK/BMAL1 complex utilizes acetyl-CoA (primarily from lactate) and short-chain acyl-CoA to enhance histone acetylation and transcriptional activity, with CREB-P providing additional support. At the Per2 promoter, CLOCK/BMAL1 and CREB-P (primarily from succinate signaling) synergistically enhance transcription, supported by short-chain acyl-CoA availability. This metabolite-mediated inter-tissue communication ensures coordinated circadian gene expression between adipose tissue and skeletal muscle. **Abbreviations:** ↑, increased; ↓, decreased; →, signaling direction or effect (multiple arrows indicate greater magnitude of effect).

The mechanism is summarized in Figure 2, illustrating how adipocyte AMPKα2 activation alters systemic metabolite profiles, which in turn regulate skeletal muscle clock genes and metabolic function. To uncover the mechanistic basis of such adipose–muscle communication, Chen et al. (2025) performed detailed molecular profiling [5]. They revealed a dramatic change in serum metabolites upon adipocyte-specific Prkaa2 knockdown, among which lactate and succinate levels showed significant changes [5]. These metabolites may act as signaling molecules that mediate adipose-to-muscle communication. The perspective of metabolites as signaling molecules is a departure from viewing them as merely metabolic intermediates.

Lactate, once considered a waste product of anaerobic glycolysis, has emerged as an important signaling molecule capable of regulating gene expression in various tissues [47]. In skeletal muscle, lactate can directly influence the expression of clock genes through multiple mechanisms. Lactate serves as a substrate for histone lactylation, a recently discovered post-translational modification that can activate gene transcription [48]. This modification may directly affect the chromatin structure at clock gene loci, particularly at BMAL1 and PER2 promoters, leading to altered circadian gene expression patterns.

Furthermore, lactate can modulate AMPK activity in muscle cells through changes in the cellular AMP/ATP ratio. When lactate is metabolized to pyruvate in muscle mitochondria, it can either enhance ATP production (if oxidative capacity is sufficient) or compete with glucose oxidation (if oxidative capacity is limited), thereby affecting local AMPK activation. This creates a feedback mechanism where adipose tissue lactate production can influence muscle AMPK activity and, consequently, muscle clock gene expression.

Succinate, an intermediate of the TCA cycle, has been demonstrated to be both a metabolic substrate and signaling molecule [49]. Extracellular succinate binds to specific G-protein-coupled receptors (GPCRs), particularly SUCNR1, and activates downstream signaling cascades that influence gene expression [50]. In the context of fat–muscle crosstalk, succinate released from adipose tissue acts on muscle cells to regulate clock gene expression through SUCNR1-mediated pathways.

The signaling cascade involves succinate binding to SUCNR1, which activates phospholipase C and increases intracellular calcium levels. Succinate-induced calcium signaling may activate CREB, influencing clock gene expression [51]. Additionally, SUCNR1 activation can modulate adenylyl cyclase activity, affecting cAMP levels and PKA activity, which in turn influences the phosphorylation status of clock proteins and their transcriptional activity.

### 5.3. Circadian–Metabolic Integration Through Acyl-CoA Species

Chen et al. (2025) identified that adipocyte-specific Prkaa2 knockout caused extensive changes in the GWAT lipidome, significantly influencing the rhythmic patterns of acyl-CoA species [5]. These lipid metabolites have the potential to function as signaling molecules or metabolic substrates that influence gene expression in distant organs. Acyl-CoA species serve as more than just metabolic intermediates; they act as allosteric regulators of various enzymes and transcription factors involved in circadian clock regulation.

Different chain-length acyl-CoA molecules can modulate the activity of acetyl-CoA carboxylase (ACC), the rate-limiting enzyme in fatty acid synthesis, which is also a direct substrate of AMPK [52]. When AMPK phosphorylates and inactivates ACC, it not only inhibits fatty acid synthesis but also alters the cellular pool of malonyl-CoA, which serves as an allosteric inhibitor of carnitine palmitoyltransferase I (CPT1). This metabolic shift affects β-oxidation rates and the production of acetyl-CoA, which is a critical substrate for histone acetylation.

The availability of acetyl-CoA for histone acetylation directly impacts chromatin structure at clock gene promoters. CLOCK protein functions as a histone acetyltransferase, and its activity is dependent on acetyl-CoA availability [53]. When adipocyte AMPKα2 signaling alters the systemic distribution of acyl-CoA species, it can affect acetyl-CoA levels in muscle cells, thereby modulating CLOCK’s acetyltransferase activity and the transcriptional output of clock genes such as PER2 and BMAL1.

Additionally, long-chain acyl-CoA species can directly interact with transcriptional regulators. Long-chain acyl-CoAs may modulate transcription factors such as SREBPs and PPARs, contributing to circadian regulation of metabolism [54]. This creates a mechanism whereby adipose tissue lipid metabolism, controlled by AMPKα2, can influence the transcriptional landscape of muscle cells through circulating acyl-CoA species.

### 5.4. Tissue-Specific Clock Synchronization

The discovery that adipocyte AMPKα2 depletion affects clock gene expression in muscle under DRF conditions reveals a sophisticated mechanism for inter-tissue circadian synchronization [5]. This synchronization is essential for coordinated metabolic responses across tissues and ensures that peripheral clocks remain aligned with feeding schedules and energy availability.

The synchronization process involves multiple temporal signals emanating from adipose tissue. The rhythmic release of adipokines such as adiponectin and leptin follows circadian patterns that are influenced by local adipose tissue clock genes, which in turn are regulated by AMPKα2 [55]. These adipokines act on muscle cells through their respective receptors (AdipoR1/R2 for adiponectin, LepR for leptin) and activate signaling pathways that can modulate clock gene expression.

Adiponectin signaling through AdipoR1 activates AMPK in muscle cells, creating a direct link between adipose tissue rhythms and muscle AMPK activity [56]. This muscle AMPK activation then feeds back on local clock gene expression through the CRY1/CRY2 phosphorylation and degradation pathway described earlier. This creates a tissue-to-tissue communication network where the phase and amplitude of circadian oscillations in one tissue can influence the circadian state of distant tissues.

The temporal coordination is further enhanced by the rhythmic production of extracellular vesicles (EVs) from adipose tissue [57]. Adipose-derived EVs may carry clock-modulating signals in a circadian manner, offering a potential mechanism for inter-tissue synchronization.

### 5.5. Integration with Exercise-Induced Signals

The fat–muscle temporal crosstalk dimensions are particularly intriguing in light of the findings of Chen et al. (2025) regarding the circadian regulation of such communication [5]. The fact that AMPK activator C29 has endurance-promoting and muscle function effects in a time-of-day-dependent manner, requiring intact adipocyte AMPKα2 signaling, means that therapeutic manipulation of fat–muscle crosstalk may be time-of-day-dependent for its effectiveness [5]. This time dependence is important for the timing of interventions and design of chronopharmacological strategies.

Exercise itself is a potent stimulus for AMPK activation in both muscle and adipose tissue, but the timing of exercise can differentially affect the circadian coupling between these tissues [58]. Morning exercise may preferentially activate muscle AMPK while having minimal effects on adipose tissue circadian rhythms, whereas evening exercise appears to coordinate AMPK activation across both tissues, leading to enhanced synchronization of their respective circadian clocks.

The exercise-induced coordination involves mechanical and humoral factors. Muscle contraction releases myokines such as IL-6, irisin, and FNDC5, which can act on adipose tissue to modulate local AMPK activity and clock gene expression [41]. Conversely, exercise-induced changes in adipose tissue metabolism, including enhanced lipolysis and altered adipokine secretion, can provide feedback signals to muscle that influence the post-exercise recovery of muscle clock genes.

This bi-directional communication ensures that the metabolic adaptations to exercise are coordinated across tissues and that the timing of these adaptations aligns with circadian rhythms in energy utilization and storage. The disruption of this coordination, as observed in adipocyte AMPKα2 knockout mice, leads to impaired exercise performance and altered metabolic flexibility, highlighting the importance of intact fat–muscle circadian communication for optimal physical function. Table 2 provides an overview of critical molecular mediators that facilitate bidirectional fat–muscle communication, detailing their functional roles and involvement in circadian regulation.

## 6. Interaction Between Dietary Timing and Exercise

The temporal synchrony of exercise and diet is the intersection of two lifestyle interventions that have robust control over metabolic health and body functions [2]. Recent studies have begun to unravel the molecular basis of this interaction, wherein temporal synchrony between exercise and feeding has been identified as the key point [59].

Chen et al. (2025) provided robust evidence of the beneficial effects of day-restricted feeding (DRF) on exercise performance in mice [5]. They demonstrated that DRF profoundly increases physical stamina, an effect mediated in part via adipocyte AMPKα2 signaling [5]. This study demonstrated that restricting feeding to the active phase (night in nocturnal mice) enhanced running stamina by modulating fat–muscle crosstalk [5]. The crucial role of adipocyte AMPKα2 was underscored by the finding that its tissue-specific knockdown ablated the DRF-induced improvement in endurance [5].

Chen et al. (2025) was particularly elegant in its study design to demonstrate the causal relationship among DRF, adipocyte AMPKα2, and exercise performance [5]. By using tissue-specific knockout models, they were able to successfully decouple the activity of adipocyte AMPKα2 from other potential confounders. Global and muscle-specific knockout mice did not show the same phenotype as adipocyte-specific knockout mice, indicating that adipose tissue plays a central role in mediating the effects of dietary timing on exercise performance.

The mechanisms underlying DRF’s beneficial effects of DRFs on exercise performance are multifaceted. Chen et al. (2025) showed that DRF enhances the rhythmic regulation of metabolic genes in adipose tissue, increasing those involved in acyl-CoA metabolism [5]. Enhanced rhythmicity may provide a maximized supply of energy substrates to the muscles during exercise. DRF also appears to improve synchronization of the adipose tissue clock with the muscle clock, providing synchronized metabolic responses to exercise requirements.

The significance of exercise timing was further supported by Savikj et al. (2022), who performed a randomized crossover trial in patients with type 2 diabetes to assess the impact of exercise timing on multi-tissue metabolome and skeletal muscle proteome profiles [7]. This study showed diverse metabolomic and proteomic changes when exercise was performed at different times of the day [7]. Specifically, they determined that post-lunch high-intensity interval training (16:45) elicited greater plasma carbohydrate changes via the pentose phosphate pathway and greater decreases in skeletal muscle lipids than morning exercise (08:00) [7].

The post-lunch training protocol also increases skeletal muscle lipoproteins and decreases mitochondrial complex III content [7]. These findings suggest that afternoon exercise is more effective for mobilizing and utilizing intramuscular lipids, which are normally elevated in patients with insulin resistance. The reduction in the content of mitochondrial complex III following afternoon exercise may reflect an adaptive response to increase mitochondrial efficiency, or a remodeling process that occurs secondary to the distinctive metabolic requirements of afternoon exercise.

Interestingly, Savikj et al. (2022) also found that the effect of time was greater for some metabolic pathways than for others [7]. For instance, the pentose phosphate pathway, which generates the NADPH required for fatty acid synthesis and forms ribose for nucleotide synthesis, showed greater activation following afternoon exercise. This indicates that the metabolic machinery that catalyzes anabolic reactions may be more responsive to exercise stimulation at certain times of day.

Understanding the molecular basis of the interaction between exercise and dietary time reveals that it is primarily mediated by the circadian control of key metabolic pathways in both adipose tissue and skeletal muscle [60]. The central clock machinery generates approximately 24 h cycles of gene expression that, in turn, regulate periodic fluctuations in protein abundance, enzyme activities, and metabolite concentrations. Temporal organization ensures that metabolic processes phase optimally to coordinate with the body’s needs during the day–night cycle.

Basse et al. (2018) demonstrated exercise-training-independent circadian rhythmicity in skeletal muscle insulin sensitivity [12]. They reported that whole-body insulin tolerance and signaling pathways that control insulin- and exercise-stimulated glucose uptake in skeletal muscles, including AKT, AMPK, and TBC1D4 phosphorylation, are circadian [12]. Rhythmicity was unaffected by exercise training, suggesting that circadian regulation of muscle metabolism is dissociable from acute responses to exercise [12].

**Figure 3:**
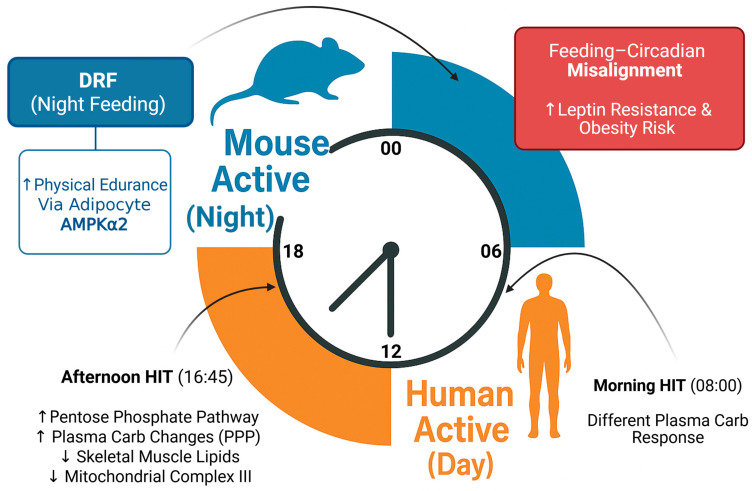
Circadian regulation of dietary timing and exercise performance.

This circadian rhythmicity independence of exercise training status is an important consequence of understanding diet timing–exercise interactions. This suggests that the beneficial effects of the synchrony of diet and exercise timing are not a consequence of heightened muscle function but rather an optimization of inter-tissue communication and metabolic coordination. This concept is visually summarized in Figure 3, which contrasts the effects of aligned versus misaligned feeding and exercise timing in nocturnal mice and diurnal humans, illustrating how synchronization enhances metabolic efficiency while misalignment increases disease risk.

Several studies have highlighted the potential harm caused by temporal desynchronization between exercise and feeding. Oishi and Hashimoto (2018) showed that nighttime feeding (day feeding in nocturnal mice) led to the development of leptin resistance, resulting in obesity and metabolic disease [11]. This supports the fact that temporal desynchronization of the feeding schedule with the inherent endogenous circadian rhythm disrupts normal metabolic homeostasis, which in turn decreases the positive impact of exercise [61].

Recent human clinical trials explored the practical implications of these results. Sutton et al. (2018) investigated the effects of time-restricted eating and exercise training in overweight adults [62]. They showed that restricting food intake to an 8 h window during the day, with added morning exercise, produced greater changes in body composition and metabolic parameters than exercise training alone. This study presents preliminary evidence that synchronizing the timing of diet and exercise can make lifestyle interventions more effective for humans.

The effect of exercise on meal timing is also influenced by personal chronotypes, which define the tendency of an individual to perform morning or evening activities. People with different chronotypes may respond differently to exercises performed at different times of day. Evening chronotypes, for instance, may have better performance and adaptation to exercise performed in the afternoon or evening, whereas morning chronotypes may value more morning exercise sessions.

This schematic contrasts the circadian activity patterns of nocturnal (mouse) and diurnal (human) organisms and highlights how temporal coordination of feeding and exercise influences metabolic outcomes. The mouse segment is based on findings from Chen et al. (2025) [5], demonstrating that day-restricted feeding (DRF) during the active (dark) phase improves endurance via adipocyte-specific Prkaa2 knockout mouse models. The human segment integrates clinical data from Savikj et al. (2022) [7], showing that afternoon high-intensity interval training (HIT) at 16:45 enhances plasma carbohydrate handling and skeletal muscle lipid metabolism more than morning HIT at 08:00 in patients with type 2 diabetes.

Additionally, Oishi and Hashimoto (2018) [11] demonstrated that feeding during the rest phase in mice induces leptin resistance and obesity, underscoring the detrimental effects of feeding–circadian misalignment. Collectively, the figure emphasizes the importance of aligning lifestyle interventions with circadian biology to optimize metabolic health.

## 7. Multi-Omics Approaches: New Insights

The use of multi-omics technologies has revolutionized our understanding of intricate biological systems and enabled unprecedented molecular mechanism descriptions of physiological and pathological processes [17]. New evidence for adipocyte AMPKα2 signaling in controlling fat–muscle communication and exercise performance has been provided by recent studies using these new analytical tools [63].

Chen et al. (2025) employed a sophisticated multi-omics approach to investigate the implications of day-restricted feeding (DRF) on fat–muscle crosstalk and adipose tissue metabolism [5]. Quantitative proteomics, lipidomics, and phosphoproteomics were employed to profile rhythmic proteins in gonadal white adipose tissue (GWAT) of mice under DRF or night-restricted feeding (NRF) [5]. Multidimensional analysis revealed adipocyte mitochondrial metabolism and the AMPKα2 pathway as significant diurnal signatures linked to daily restricted feeding (DRF) [5].

Proteomic analysis showed that DRF promoted the rhythmic expression of key metabolic enzymes in the adipose tissue. Specifically, proteins involved in fatty acid β-oxidation, TCA cycle, and oxidative phosphorylation showed higher circadian oscillations under DRF conditions. The amplitude of the oscillations was significantly higher than that under NRF, suggesting that DRF strengthens the molecular clock machinery in adipose tissue.

Lipidomic profiling provided further information on metabolic changes induced by DRF. Chen et al. (2025) identified over 500 lipid species in GWAT, most of which showed impressive circadian fluctuation [5]. After DRF treatment, the circadian oscillations of neutral lipids, phospholipids, and sphingolipids were enhanced and enriched, with a particular enrichment of acyl-CoA species involved in fatty acid metabolism. The disruption of rhythmicity in these lipid species after adipocyte-specific Prkaa2 knockdown validated the pivotal role of AMPKα2 in synchronizing lipid metabolism with circadian rhythms.

Phosphoproteomics revealed that dynamic protein phosphorylation was regulated upon exposure to DRF. This approach identified numerous rhythmically fluctuating phosphorylation sites, most of which were located on proteins of the metabolic pathway. AMPK showed rhythmic phosphorylation at its activating position (Thr172) and peak phosphorylation at the active feeding time in the presence of DRF.

The integration of these omics datasets allowed Chen et al. (2025) [5] to establish a systems-level image of the molecular mechanisms underlying adipose tissue adaptation to different feeding regimens [64]. Computational approaches have been used to identify key regulatory networks and predict novel interactions among metabolic pathways. This systems-level analysis revealed AMPKα2 as a core hub that converges various metabolic signals and regulates cellular responses to nutrient availability.

The ability of single-cell analysis to decipher cellular heterogeneity of muscle and adipose tissues was established by Yang et al. (2022) [6]. Their study utilized single-cell RNA sequencing (scRNA-seq) to explore the obesity–exercise axis within adipose and muscle tissues, uncovering that mesenchymal stem cells (MSCs) play a key role in the tissue remodeling processes influenced by obesity and exercise [6]. This technique has deciphered heterogeneous cell population responses to exercise and obesity, illustrating the complexity of intercellular and intertissue signal networks [57].

Single-cell analysis identified cell populations in the muscle and fat tissues that responded differently to metabolic stress. Yang et al. (2022) identified subpopulations of adipocytes, immune cells, and stromal cells with characteristic gene expression profiles in fat tissue [6]. Conversely, MSCs display striking changes in circadian rhythms and expression of extracellular matrix remodeling genes following exercise and obesity. This cell-level examination uncovered aspects that would have gone unnoticed if the tissues had been examined in bulk.

In muscle tissue, the study showed the presence of different types of muscle fibers with different metabolic characteristics. Slow- and fast-twitch muscles were found to adapt differentially to exercise training and showed more changes in mitochondrial and metabolic genes in the slow-twitch type. Satellite cells, or the muscle stem cells, were also found to be important mediators of exercise-induced muscle adaptation, as indicated by the analysis.

The application of metabolomics and proteomics analyses to human material has been supported by Savikj et al. (2022), who undertook a multi-tissue-wide metabolome and skeletal muscle-wide proteome study of how exercise timing in patients with type 2 diabetes was affected [7]. This study applied broad-scale, untargeted metabolomic profiling of blood, skeletal muscle, subcutaneous adipose tissue, and skeletal muscle proteins using proteomic profiling of samples [7].

The metabolomics study detected over 1000 metabolites in the three tissues, as well as hundreds of time-of-day-dependent responses to exercise. This study detected the characteristic patterns of amino acids, lipids, and carbohydrates in the blood that varied according to the timing of exercise. The pentose phosphate pathway was most significantly affected by afternoon exercise, with elevated levels of pathway intermediates and end products.

In the skeletal muscle, metabolomic analysis revealed differences in lipid composition and amino acid concentrations between afternoon and morning exercise protocols. Proteomic analysis revealed differential expression of proteins involved in mitochondrial function, lipid metabolism, and protein synthesis. Remarkably, proteins involved in the unfolded protein response and endoplasmic reticulum stress were upregulated after afternoon exercise, revealing that time-of-day differences in exercise could result in differential cellular stress responses.

The integrated approach employed by Savikj et al. (2022) revealed coordinated alterations in protein classes and metabolic pathways during exercise with time-of-day-specific profiles [7]. The ability to simultaneously quantify various tissues and molecular classes provides a broader view of systemic responses to exercise and highlights the role played by temporal considerations in these responses [65]. The study demonstrated that an identical exercise protocol generates vastly different molecular signatures depending on when it is performed, demonstrating the complexity of exercise responses.

Multi-omics approaches are important for identifying biomarkers and therapeutic targets. The high-resolution molecular details offered by these platforms have revealed slight but important differences that are not necessarily apparent from standard measures of physiology. For example, Chen et al. (2025) identified specific acyl-CoA species with distinct circadian patterns under different feeding conditions [5]. These lipid metabolites may serve as biomarkers of circadian metabolic health or as therapeutic targets.

The temporal information provided by multi-omics is particularly valuable for understanding circadian metabolism [66]. Chen et al. (2025) and Savikj et al. (2022) both incorporated temporal aspects into their research designs, allowing them to track the dynamic evolution of molecular profiles over time or after interventions at different times of day [5,7]. This temporal dimension is necessary to understand the circadian aspects of metabolic regulation and the interactions between feeding time, exercise time, and endogenous circadian rhythms [67].

Recent developments in computational biology have enhanced the interpretation of multi-omics data. Machine learning algorithms are capable of identifying sophisticated patterns in high-dimensional data that human examinations cannot identify. Network analysis can reveal new relationships between different molecular species and their pathways. Such computational processes are vital for revealing significant biological information from the vast amounts of data generated by multi-omics strategies.

Despite these advances, multi-omics technologies face important spatial resolution limitations that constrain our understanding of metabolic processes. Current bulk tissue analysis approaches cannot capture single-cell metabolic heterogeneity within tissues, potentially missing critical cell-type-specific responses to temporal interventions [68]. For instance, while Chen et al. (2025) demonstrated adipocyte AMPKα2’s role in fat–muscle crosstalk, the specific adipocyte subpopulations mediating these effects remain unclear due to bulk tissue profiling limitations [5]. Similarly, metabolomic analyses at the single-cell level remain technically challenging, limiting our ability to understand metabolic flux variations among individual cells within the same tissue [69].

Emerging spatial transcriptomics technologies offer promising solutions to these limitations by preserving spatial context while providing molecular resolution [70]. Recent applications of spatial transcriptomics to adipose tissue have revealed previously unrecognized spatial organization of metabolic gene expression, with distinct microenvironments showing different responses to nutritional and exercise stimuli [71]. Future integration of spatial multi-omics approaches with temporal intervention studies could provide unprecedented insights into how circadian signals propagate through tissue-specific cellular networks and influence fat–muscle communication [72].

Other forms of biological data, such as functional genomics data and phenomenological measurements, are integrated in everyday practice. A systems biology style such as this provides a far better picture of the biological processes being studied and results in new mechanistic insights. Integration of transcriptomic data with metabolomic data provides a means to track the flux from genes to proteins to metabolites, and thus reach pathway-level comprehension of metabolic control.

## 8. Clinical Implications: Temporal Intervention Strategies

Deeper insight into the molecular mechanisms governing the interaction between meal time, adipocyte AMPKα2 activity, and physical activity function carries significant clinical implications, particularly for tailoring temporal therapies for metabolic disorders [73]. New experimental findings provide a platform to expand such understanding into effective clinical schemes, but still fall short of definitively establishing such programs in human populations [74].

Chen et al.’s (2025) research shows that coordination of feeding with the active phase, like in day-restricted feeding (DRF), enhances exercise performance by optimizing adipocyte AMPKα2 signaling and fat–muscle crosstalk [5]. These findings have direct therapeutic implications for diseases associated with impaired physical performance, including obesity, type 2 diabetes, and sarcopenia [60]. By timing food consumption during the activity period, individuals may be able to enhance the physical and metabolic consequences of exercise [61]. It is a relatively inexpensive and simple intervention that can be easily incorporated into existing lifestyle modification schemes [75].

Time-restricted eating (TRE) has emerged as a topic of significant clinical interest. Several human trials have demonstrated that restricting food intake to an 8–12 h window in the day can enhance metabolic health, regardless of caloric restriction. For instance, Sutton et al. (2018) found that early time-restricted feeding (consuming meals only from 8:00 AM to 2:00 PM) increased insulin sensitivity and β-cell function in prediabetic men [62]. These findings provide evidence for the clinical applicability of temporal feeding regimens.

The optimal application of DRF or TRE is subject to the consideration of individual factors, such as chronotype, lifestyle, and disease state. Healthcare practitioners must consider these factors when prescribing temporal feeding schedules. For example, individuals working night shifts may require different feeding schedules than those with standard working hours. Patients with diabetes who require medication after food intake may also require modified TRE protocols.

However, implementation of temporal interventions faces significant compliance challenges, particularly among populations with irregular schedules. Shift workers represent a critical population where circadian misalignment contributes substantially to metabolic dysfunction, yet implementing DRF protocols presents unique barriers [55]. Night-shift workers experience inverted sleep–wake cycles and altered meal timing patterns that conflict with conventional TRE recommendations [76]. Studies have shown that shift workers attempting standard TRE protocols often experience increased fatigue, impaired work performance, and poor adherence rates due to social and occupational constraints [77].

Digital health solutions offer promising approaches to address these compliance challenges. Wearable monitoring devices equipped with circadian rhythm tracking capabilities can provide personalized guidance for implementing temporal interventions [78]. These devices can monitor sleep–wake patterns, physical activity, and even metabolic markers to optimize individual timing protocols [79]. Smartphone applications integrated with wearable data can deliver real-time coaching and adaptive recommendations that adjust feeding and exercise windows based on individual circadian patterns and lifestyle constraints [80].

For shift workers specifically, digital platforms could implement modified DRF protocols that align with individual work schedules rather than conventional day–night cycles. Machine learning algorithms could analyze patterns of sleep, activity, and metabolic responses to develop personalized intervention timing that maximizes metabolic benefits while maintaining work performance and social functioning [81]. Virtual coaching systems could provide support during challenging transition periods and help maintain adherence through behavioral reinforcement strategies [82].

Savikj et al. (2022) emphasized the potential maximal effects of exercise time optimization on type 2 diabetes [7]. Their observation that exercise in the afternoon (16:45) had differential impacts on plasma carbohydrates and skeletal muscle lipids compared to morning exercise (08:00) suggests that therapeutic effectiveness may be maximized by tailoring exercise times to suit individual metabolic goals [7]. For example, individuals seeking to increase muscle insulin sensitivity or reduce intramuscular lipid content may benefit from afternoon exercise training [83]. This individualized exercise prescription is a promising avenue for the optimal treatment of type 2 diabetes and other metabolic disturbances [84].

The clinical use of timed exercise therapy requires consideration of various factors such as patient desire, working hours, and other medications. Clinicians may need to work with patients to determine the most feasible exercise timing that complements circadian inclinations and lifestyle restrictions. Additionally, the coordination of meals and exercise timing should be considered to realize the synergistic action of these interventions.

The discovery that the AMPK activator C29 enhances endurance and muscle performance in a time-of-day specific manner, contingent upon intact adipocyte AMPKα2 signaling, carries significant implications for chronopharmacological approaches aimed at optimizing exercise performance and treating metabolic diseases [5]. This suggests that by administering AMPK-activating drugs at specific times of the day, it may be possible to maximize their therapeutic benefits while concurrently minimizing adverse effects [85]. This principle of time-based drug administration, known as chronopharmacology, has garnered increasing attention and could represent a crucial advancement in the management of various conditions, including metabolic disorders [86].

Current chronopharmacological investigations also seek to ascertain the most propitious drug timing for peak performance based on the fluctuating rhythms of drug metabolite formation and drug sensitivity in the target organs. For AMPK activators, dosing time might indeed be a determinant of the maximum beneficial and minimum side effect potential of the drug. Long-acting dosing solutions or time-scheduling drug administration schemes may be utilized to apply the principles of chronopharmacology clinically.

The finding that skeletal muscle insulin sensitivity exists in circadian rhythmicity, as shown by Basse et al. (2018), has implications for the timing of glucose monitoring and insulin administration in patients with diabetes [12]. By considering the natural variation in insulin sensitivity throughout the day, clinicians can adjust insulin dosing regimens to more closely match the body’s metabolic needs, potentially reducing the risk of hypoglycemia and improving glycemic control [87]. Similarly, the timing of oral antidiabetic agents may be optimized during periods of increased insulin resistance, thereby maximizing their efficacy [88].

The current continuous glucose monitoring (CGM) technology provides precise feedback on glucose profiles throughout the day and can potentially be used to optimize the timing of medications and lifestyle interventions. The integration of CGM with information on the circadian rhythms of insulin sensitivity can lead to more precise and customized diabetes management strategies.

The use of integrated temporal intervention programs could involve the coordination of more than one intervention strategy such as time-restricted feeding, time-based exercise sessions, or chronopharmacological interventions. Such multicomponent programs should be carefully implemented and tracked to achieve maximum effectiveness. Table 3 summarizes representative clinical applications of temporal intervention strategies, including their mechanisms of action, potential metabolic benefits, target conditions, and practical considerations for implementation. Health informatics technologies such as smartphone apps and wearable devices can assist in implementing and tracking multi-component interventions.

Smart wearable devices can continuously monitor physiological parameters including heart rate variability, skin temperature, and activity patterns to assess circadian rhythm status and intervention compliance [89]. Advanced algorithms can detect circadian disruption early and recommend adjustments to temporal intervention protocols [90]. Integration with electronic health records allows healthcare providers to monitor patient progress remotely and make data-driven adjustments to treatment plans [91].

Concordance and patient education are central elements in the effectiveness of temporal intervention methods. The rationale and practical recommendations for implementation must be clearly explained to patients by the treating physician. Producing educational materials and support tools plays a central role in the universal application of these strategies.

Future research should focus on establishing personalized temporal intervention approaches based on individual chronotypes, genetic predispositions, and metabolic status. Circadian health biomarkers and treatment responses can be employed to guide the optimization of temporal interventions for individual patients. Long-term studies are necessary to ascertain the safety and efficacy of such interventions over extended durations.

## 9. Conclusions

This review synthesizes recent experimental data, indicating that adipocyte AMPKα2 signaling critically regulates circadian fat–muscle communication, thereby improving exercise performance. The landmark article by Chen et al. (2025) [5] revealed day-restricted feeding to optimize such communication through adipocyte-specific AMPKα2 activation, and corroborative studies by Yang et al. (2022), Savikj et al. (2022), Oishi and Hashimoto (2018), and Basse et al. (2018) have provided evidence for the temporal coordination function in metabolism [6,7,11,12].

Exercise and food timing in relation to endogenous circadian cycles have become major regulators that maximize their therapeutic impact [92]. Continuous application of these technologies will progressively uncover the dynamic regulation of metabolism and exercise physiology [93]. Day-restricted eating enhances exercise function through adipocyte AMPKα2-mediated communication between muscle and fat, while exercise at specific times of day evokes differential metabolic adaptations. Translation of these findings into a clinical setting holds huge potential for the provision of better and tailored treatment of metabolic diseases and exercise prescriptions [94].

Multi-omics approaches have shed light on the complex molecular mechanisms underlying these events. Time-restricted feeding, exercise regimens timed with the circadian rhythm, and chronopharmacological stimulation of AMPK are likely effective interventions that can be readily added to existing treatment strategies [95]. Highly advanced approaches such as proteomics, lipidomics, phosphoproteomics, and single-cell RNA sequencing have provided unparalleled insights into the cellular and molecular basis of fat–muscle crosstalk, revealing novel regulatory networks and therapeutic targets. These findings highlight the importance of considering the timing of lifestyle interventions in preventing and treating metabolic diseases.

The integration of timing in metabolic medicine represents not a gradual advance but rather a fundamental reconsideration of the prevention and treatment of metabolic diseases. Future metabolic medicine will likely involve sophisticated temporal intervention approaches that are personalized, based on individual chronotypes, genetic profiles, and metabolic phenotypes. Digital health technologies, including wearable monitoring devices and smartphone applications, offer promising solutions to implementation challenges, particularly for populations with irregular schedules such as shift workers.

However, significant challenges remain in translating these discoveries into widespread clinical practice. Compliance barriers, particularly among shift workers and individuals with irregular schedules, require innovative digital solutions and personalized intervention protocols. Multi-omics technologies, while powerful, face spatial resolution limitations that future spatial transcriptomics approaches may address. Despite the strengths of current multi-omics approaches, their limited spatial resolution hinders the detection of cell-type-specific metabolic responses within complex tissues. Emerging spatial transcriptomics technologies offer a promising solution by preserving tissue architecture while capturing gene expression patterns, and hold great potential for advancing our understanding of how circadian signals coordinate fat–muscle communication at the cellular level.

The insight that “when” we have a meal or exercise is as important as “what” we eat or “how much” we exercise represents a conceptual leap in the direction of wellness and health. Such a temporal perspective generates new intervention and optimization possibilities that were previously undervalued. By tapping into the potential of circadian rhythms and optimizing the timing of activities, we can unlock new ranges of metabolic health and physical function. The journey to such a future will be one of the continued interactions among basic scientists, clinicians, and technologists to bring these exciting discoveries into effective interventions that benefit human health.

## Figures and Tables

**Table 1:** Summary of key studies on temporal aspects of fat–muscle crosstalk.

Study	Model	Intervention	Key Findings	Significance
Chen et al. (2025) [5]	Mouse (GWAT-specific Prkaa2 knockout)	Day-restricted feeding (DRF), AMPK activator (C29)	Adipocyte AMPKα2 regulates adipose metabolism, modulates muscle clock	Adipose acts as circadian regulator of muscle
Yang et al. (2022) [6]	Mouse (single-cell analysis)	Obesity + exercise	Circadian genes altered in stromal cells	Fat–muscle circadian crosstalk
Savikj et al. (2022) [7]	Human (T2DM patients)	Morning vs. afternoon exercise	PM exercise ↑ mitochondria and lipids	Exercise timing impacts metabolism
Mancilla et al. (2021) [8]	Human (metabolically compromised)	Morning vs. afternoon exercise training	PM training ↑ insulin sensitivity and performance	Superior metabolic benefits from PM training
Ezagouri et al. (2019) [10]	Mouse and Human	Exercise at different times	Time-dependent exercise capacity via PER1/2	Circadian control of exercise performance
Oishi & Hashimoto (2018) [11]	Mouse	Time-restricted feeding during resting period	Leptin resistance, obesity	Feeding timing affects metabolism
Basse et al. (2018) [12]	Human	Circadian rhythm vs. exercise training	Circadian insulin sensitivity	Clock-regulated sensitivity independent of exercise

Abbreviations: ↑, increased.

**Table 2:** Molecular mediators of fat–muscle crosstalk.

Molecule	Origin	Target Tissue	Function	Circadian Regulation	Molecular Mechanism
AMPKα2	Adipose tissue	Adipose, Skeletal muscle	Energy sensor, regulates metabolic homeostasis, controls muscle clock genes	Yes	Activates downstream targets like ACC and PGC-1α; regulates Bmal1 and Per2 expression [5]
Lactate	Muscle, Adipose	Liver, Muscle, Adipose	Energy substrate, signaling molecule, gene expression regulation	Yes	Binds to GPR81; regulates gene expression via HIF-1α and CREB pathways [29]
Succinate	Multiple tissues	Liver, Muscle, Adipose	TCA cycle intermediate, signaling molecule	Yes	Binds to SUCNR1 receptor; activates MAPK signaling and gene transcription [30]
Irisin	Muscle	White Adipose tissue	Promotes browning of white adipose tissue, enhances thermogenesis	Unclear	Derived from FNDC5 cleavage; activates UCP1 expression via PGC-1α pathway [33]
IL-6	Muscle, Adipose	Liver, Muscle, Adipose, Immune cells	Pro/anti-inflammatory cytokine, regulates glucose metabolism	Partial	Activates JAK/STAT3 and AMPK pathways; enhances glucose uptake in muscle [32]
Adiponectin	Adipose tissue	Muscle, Liver	Enhances insulin sensitivity, fatty acid oxidation	Yes	Activates AMPK and PPARα pathways; increases GLUT4 translocation [24]
Leptin	Adipose tissue	CNS, Muscle	Regulates energy balance, influences muscle metabolism	Yes	Binds to leptin receptor; activates JAK2/STAT3 and PI3K signaling [24]
Acyl-CoA	Multiple tissues	Liver, Muscle, Adipose	Metabolic intermediate, involved in fatty acid metabolism	Yes	Serves as substrate for β-oxidation; regulates CPT1 and energy flux [25]

**Table 3:** Clinical applications of temporal intervention strategies.

Intervention	Mechanism	Potential Benefits	Target Conditions	Implementation Considerations
Time-restricted eating (TRE)	Aligns feeding with active phase; optimizes adipocyte AMPKα2 signaling	Enhanced exercise performance; improved adipose–muscle communication; metabolic benefits	Obesity, Type 2 diabetes, sarcopenia	8–12 h eating window during active phase; consistent daily timing essential
Time-optimized exercise	Utilizes circadian variations in metabolic responses to exercise stimuli	Improved lipid metabolism, mitochondrial function, insulin sensitivity	Type 2 diabetes, metabolic syndrome	Afternoon (16:00–18:00) sessions may be superior for certain outcomes
Chronopharma-cological AMPK targeting	Time-specific activation of AMPK pathways via pharmacological agents	Enhanced muscle endurance, metabolic regulation, reduced side effects	Metabolic disorders, physical fatigue syndromes	Requires synchronized drug timing; dependent on functional AMPKα2 in adipose
Time-optimized antidiabetic medication	Aligns drug action with peak insulin resistance periods	Greater glycemic stability; higher medication efficacy	Type 2 diabetes	Consider circadian rhythm of insulin sensitivity; personalized schedules recommended
Digital-assisted temporal interventions	Wearable monitoring with real-time coaching and adaptive recommendations	Improved compliance; personalized protocols; remote monitoring capabilities	Complex metabolic disorders, shift work-related dysfunction	Integration with healthcare systems; requires digital literacy
Integrated temporal approach	Combines timing of feeding, exercise, and medication for synergistic impact	Comprehensive metabolic improvement; potential adherence boost	Complex metabolic disorders, lifestyle disease management	Needs coordination across interventions; potential for digital support tools
